# Supplementary material for: An Ecological Study Relating the SARS-CoV-2 Epidemiology with Health-Related, Socio-Demographic, and Geographical Characteristics in South Tyrol (Italy)
Source: Int J Environ Res Public Health. 2024 Nov 30;21(12):1604. doi: 10.3390/ijerph21121604 (PMC11675570; doi:10.3390/ijerph21121604)
Supplement: Supplementary file 1 [file ijerph-21-01604-s001.zip › ijerph-3305901-supplementary.pdf]

Supplementary Material

Supplementary Table S1. The 118 municipalities in South Tyrol grouped in the 20 districts and 4 health sections to which they belong.

Section	District	Municipality
**Bolzano**	Bassa Atesina	Aldino, Anterivo, Cortaccia, Cortina, Egna, Magre', Montagna, Ora, Salorno, Termeno, Trodena Nel Parco Naturale
	Bolzano	Bolzano
	Laives - Bronzolo - Vadena	Bronzolo, Laives, Vadena
	Oltradige	Andriano, Appiano, Caldaro, Nalles, Terlano
	Salto - Val Sarentino - Renon	Meltina, Renon, San Genesio, Atesino, Sarentino
	Val D'ega - Sciliar	Castelrotto, Cornedo, All'isarco, Fie' Allo Sciliar, Nova Levante, Nova Ponente, Tires
	Val Gardena	Ortisei, Santa Cristina Valgardena, Selva Di Val Gardena
**Bressanone**	Alta Valle Isarco	Brennero, Campo Di Trens, Fortezza, Racines, Val Di Vizze, Vipiteno
	Bressanone Circondario	Bressanone, Luson, Naz-Sciaves, Rio Di Pusteria, Rodengo, Vandoies, Varna
	Chiusa Circondario	Barbiano, Chiusa, Funes, Laion, Ponte Gardena, Villandro, Velturno
**Brunico**	Alta Val Pusteria	Braies, Dobbiaco, Monguelfo-Tesido, San Candido, Sesto Valle Di Casies, Villabassa
	Brunico Circondario	Brunico, Chienes, Falzes, Gais, Perca, Rasun Anterselva, San Lorenzo Di Sebato, Terento, Valdaora
	Tures Aurina	Campo Tures, Predoi, Selva Dei Molini, Valle Aurina
	Val Badia	Badia, Corvara In Badia, Marebbe, San Martino In Badia, La Valle
**Merano**	Alta Val Venosta	Curon Venosta, Glorenza, Malles Venosta, Prato Allo Stelvio, Sluderno, Stelvio, Tubre
	Lana Circondario	Cermes, Gargazzone, Lana, Lauregno, Postal, Proves, San Pancrazio, Tesimo, Ultimo, Senale-San Felice
	Media Val Venosta	Castelbello-Ciardes, Laces, Lasa, Martello, Silandro
	Merano Circondario	Avelengo, Caines, Lagundo, Marlengo, Merano, Rifiano, Scena, Tirolo, Verano
	Naturno Circondario	Naturno, Parcines, Plaus, Senales
	Val Passiria	Moso In Passiria, San Leonardo In Passiria, San Martino In Passiria

Supplementary Table S2. Quantitative variables representing the geo-morphological and social characteristics of each district, health section and of the whole province.

Districts Characteristic	Demographics		Social Dynamics		Health Services			Geography	
	Inhabitants	Population Density (people/km^2)	Average Salary (€)	Winter tourists	Primary series coverage	Booster coverage	Pharmacies/ km^2	Surface (km^2)	Average Altitude (m)
Overall	20259 (15420 - 26956)	56.4 (29.3 - 90.6)	27664.8 (26689.9 - 28334.2)	491182 (230822 - 932245)	69.5 (67.7 - 71.9)	44.5 (43 - 46.2)	0.01 (0.01 - 0.02)	414.9 (241.1 - 522.0)	960.7 (729.5 - 1067.7)
Bassa Atesina	25667	104.0	26760.2	166879	69.5	45.4	0.02	247	636.9
Bolzano	107467	2055.0	25653.0	286737	74.8	51	0.48	52	262.0
Laives - Bronzolo - Vadena	22063	486.3	26479.0	78618	74.5	49.4	0.11	45	248.5
Oltradige	30773	214.7	28945.4	242744	72.9	48.9	0.03	143	384.3
Salto - Val Sarentino - Renon	19879	38.6	28011.0	190997	70.2	43.6	0.01	514	1032.0
Val D'ega - Sciliar	21199	52.1	28330.0	1159421	68.9	43.4	0.01	407	1057.8
Val Gardena	9663	86.2	27821.3	1332732	66.0	36.6	0.03	112	1453.9
Bolzano	22063 (20539 - 28220)	104.0 (69.1 - 350.5)	27821.3 (26619.6 - 28170.5)	242744 (178938 - 723079)	70.2 (69.2 - 73.7)	45.3 (43.5 - 49.2)	0.03 (0.02 - 0.07)	143.3 (82.2 - 326.8)	636.9 (323.1 - 1044.9)
Alta Val Venosta	16052	22.0	26146.1	656022	67.1	42.7	0.01	729	1232.1
Lana Circondario	25684	60.7	27602.8	253155	66.1	40.6	0.01	423	972.4
Media Val Venosta	18864	37.5	25976.8	114396	69.5	45.8	0.01	503	894.6
Merano Circondario	57956	259.1	27656.7	902476	70.6	46.2	0.05	224	655.3
Naturno Circondario	11886	21.8	27562.8	326343	67.7	43.6	0.01	545	1041.2
Val Passiria	8850	28.5	25591.0	212505	57.1	30.9	0.01	311	877.1
Merano	17458 (12927 - 23979)	33 (23.6 - 54.9)	26854.4 (26019.1 - 27592.8)	289749 (222667 - 573602)	67.4 (66.4 - 69.1)	43.2 (41.1 - 45.2)	0.01 (0.01 - 0.01)	463.2 (338.9 - 534.3)	933.5 (881.5 – 1024.0)
Alta Valle Isarco	20639	32.0	27433.0	658987	72.8	48.5	0.01	646	962.6
Bressanone Circondario	40240	85.7	28538.4	862227	70.3	46.2	0.02	470	754.2
Chiusa Circondario	17333	65.1	27964.3	236928	72.0	44.7	0.02	266	907.5
Bressanone	20639 (18986 - 30439)	65.1 (48.5 - 75.4)	27964.3 (27698.6 - 28251.4)	658987 (447957 - 760607)	72.0 (71.1 - 72.4)	46.2 (45.4 - 47.3)	0.02 (0.01 - 0.02)	469.7 (368.0 - 557.8)	907.5 (830.8 – 935.0)
Alta Val Pusteria	16213	29.5	27673.0	1021554	71.8	45.8	0.01	550	1217.6
Brunico Circondario	39469	84.4	29132.4	1379386	68.9	44.3	0.02	467	958.8
Tures Aurina	13523	24.8	28844.0	735353	67.5	42.5	0.00	544	1097.3
Val Badia	11492	28.8	28346.6	1742839	68.9	43.1	0.01	399	1296.9
Brunico	14868 (13015 - 22027)	29.2 (27.8 - 43.2)	28595.3 (28178.2 - 28916.1)	1200470 (950004 - 1470249)	68.9 (68.6 - 69.7)	43.7 (43 - 44.7)	0.01 (0.01 - 0.01)	505.9 (450.3 - 545.7)	1157.5 (1062.7 - 1237.4)

Values are represented in numbers for districts, and median (IQR) for health sections and for the whole province.

Supplementary Table S3. Qualitative variables representing the geo-morphological and health-service related characteristics of each district, health section and of the whole province.

	Health Services		Geography			
	Big hospitals presence	Small hospitals presence	Main cities district	Rural/ Towns district	Bordering with other countries	Bordering with other Italian Regions
Overall	4 (20.0%)	3 (15.0%)	4 (20.0%)	16 (80.0%)	4 (20.0%)	7 (35.0%)
Bassa Atesina				x		x
Bolzano	x		x			
Laives - Bronzolo - Vadena				x		
Oltradige				x		x
Salto - Val Sarentino - Renon				x		
Val D'ega - Sciliar				x		x
Val Gardena				x		x
Bolzano	1 (14.3%)	0 (0.0%)	1 (14.3%)	6 (85.7%)	0 (0.0%)	4 (57.1%)
Alta Val Venosta				x	x	
Lana Circondario				x		x
Media Val Venosta		x		x		
Merano Circondario	x		x			
Naturno Circondario				x		
Val Passiria				x		
Merano	1 (16.7%)	1 (16.7%)	1 (16.7%)	5 (83.3%)	1 (16.7%)	1 (16.7%)
Alta Valle Isarco		x		x	x	
Bressanone Circondario	x		x			
Chiusa Circondario				x		
Bressanone	1 (33.3%)	1 (33.3%)	1 (33.3%)	2 (66.67%)	1 (33.3%)	0 (0.0%)
Alta Val Pusteria		x		x	x	x
Brunico Circondario	x		x		x	
Tures Aurina				x		
Val Badia				x		x
Brunico	1 (25.0%)	1 (25.0%)	1 (25.0%)	3 (75.0%)	2 (50.0%)	2 (50.0%)

"X" represents the presence of that characteristic for districts, while sum (% of districts with that characteristic) are reported for health sections and the whole province.

Supplementary Table S4. Distribution of outcomes in each district, health section and in the whole province.

Health Section	Sanitary District	Positive Cases Detected	Incidence*	Hospitalisation Rate*	ICU[a] Admission Rate*	Mortality*	Hospitalisation Rate**	ICU[a] Admission Rate**	Case fatality rate**
Overall		3566 (2868 - 4994)	18090 (17078 - 19434)	162 (133 - 181)	15 (4 - 23)	35 (32 - 43)	93 (74 - 104)	7 (3 - 13)	19 (19 - 23)
	Bassa Atesina	3874	17115	90	4	39	59	3	26
	Bolzano	16983	15803	177	4	36	112	2	23
	Laives - Bronzolo - Vadena	3776	17115	168	0	32	98	0	19
	Oltradige	5061	16446	123	0	26	75	0	16
	Salto - Val Sarentino - Renon	3452	17365	96	0	10	55	0	6
	Val D'ega - Sciliar	3695	17430	132	5	33	76	3	19
	Val Gardena	2125	21991	93	0	10	42	0	5
Bolzano		3776 (3574 - 4468)	17115 (16125 - 17398)	123 (94 - 150)	0 (0 - 4)	32 (18 - 35)	75 (57 - 87)	0 (0 - 2)	19 (11 - 21)
	Alta Val Venosta	3218	20047	206	12	56	103	6	28
	Lana Circondario	4972	19358	164	19	43	84	10	22
	Media Val Venosta	3201	16969	159	5	53	94	3	31
	Merano Circondario	9338	16112	176	22	31	109	14	19
	Naturno Circondario	2069	17407	160	34	34	92	19	19
	Val Passiria	1740	19661	192	56	45	98	29	23
Merano		3210 (2352 - 4534)	18383 (17078 - 19585)	170 (161 - 188)	21 (14 - 31)	44 (36 - 51)	96 (92 - 101)	12 (7 - 18)	23 (20 - 27)
	Alta Valle Isarco	3679	17825	237	5	34	133	3	19
	Bressanone Circondario	7438	18484	221	17	35	120	9	19
	Chiusa Circondario	3222	18589	133	23	35	71	12	19
Bressanone		3679 (3451 - 5559)	18484 (18155 - 18536)	221 (177 - 229)	17 (11 - 20)	35 (34 - 35)	120 (96 - 126)	9 (6 - 11)	19 (19 - 19)
	Alta Val Pusteria	2996	18479	160	25	19	87	13	10
	Brunico Circondario	7968	20188	256	48	38	127	24	19
	Tures Aurina	2482	18354	177	44	52	97	24	28
	Val Badia	2353	20475	139	17	44	68	8	21
Brunico	*Median (IQR)*	2739 (2450 - 4239)	19333 (18448 - 20260)	169 (155 - 197)	35 (23 - 45)	41 (33 - 46)	92 (82 - 104)	19 (12 - 24)	20 (17 - 23)

Values are represented in numbers for districts, and median (IQR) for health sections and for the whole province.
**Every 100000 inhabitants; **every 10000 Cases. [a]Intensive Care Unit.*

Supplementary Table S5. Collinearity diagnoses between variables analysed in the two linear regressions.

	Collinearity Statistics	
Linear Regression on Incidence	Tolerance	VIF
Average Altitude	0.549	1.82
Primary Series Coverage	0.819	1.22
Winter Tourism	0.677	1.48
Population Density	0.563	1.78
Linear Regression on Hospital Admissions	Tolerance	VIF
Booster Coverage	0.892	1.121
Hospital Presence and Size	0.849	1.178
Bordering with other Italian Regions	0.925	1.082
Bordering with other countries	0.853	1.173
